# Peer Review of “A Framework for Modeling, Analyzing, and Decision-Making in Disease Spread Dynamics and Medicine/Vaccine Distribution (Preprint)”

**DOI:** 10.2196/64810

**Published:** 2024-09-19

**Authors:** Vanessa Fairhurst, Eustache Muteba Ayumba, Femi Qudus Arogundade, Ghadah Alnooh, Olajumoke Oladoyin, Raheel Allana, Fabien Hagenimana, Cecilia Okusi, Emmanuel Adamolekun

**Affiliations:** 1 PREreview, Oxford, United Kingdom; 2 International Medical Informatics Association, Kinshasa, Congo; 3 ASAPbio, Ibadan, Nigeria; 4 University of Sheffield, Sheffield, United Kingdom; 5 University of Texas Health Science Center at Houston, Houston, TX, United States; 6 Aga Khan University, Karachi, Pakistan; 7 University of Oxford, Oxford, United Kingdom; 8 Helix Biogen Research Institute, Ogbomosho, Nigeria

**Keywords:** COVID-19, disease spread, vaccine distribution, simulation, simulation framework

*This is a peer-review report submitted for the preprint “A Framework for Modeling, Analyzing, and Decision-Making in Disease Spread Dynamics and Medicine/Vaccine Distribution.”*

This review is the result of a virtual collaborative live review discussion organized and hosted by PREreview and JMIR Publications. The discussion was joined by 15 people: 1 author, 2 facilitators, 2 members of the JMIR Publications team, and 10 live review participants. Priti Singh and Arya Rahgozar wished to be recognized for their participation in the live review discussion, even though they have not contributed to authoring the review below. We thank all participants who contributed to the discussion and made it possible for us to provide feedback on this preprint.

## Summary

The study [1] introduces a flexible framework designed to model the dynamics of disease spread and the distribution of medicine and vaccines during epidemics and pandemics. Inspired by historical events like the Spanish flu and modern theories such as central place theory, the framework incorporates agent-based modeling to simulate intricate interactions within diverse population centers.

Central place theory, formulated by Walter Christaller, serves as the basis for arranging populations spatially, explaining how human settlements are distributed across regions. Agent-based modeling, on the other hand, enables the simulation of autonomous agents’ behaviors and interactions within the environment, predicting complex group dynamics and emergent behaviors.

During the development of the framework, certain simplifications were made to enhance simplicity and adaptability. The framework was implemented using JavaScript, HTML, and CSS for visualization, with the capability for customization through JSON parameter files. The simulation methodology guides users through the setup and execution of simulations for various scenarios, facilitating the observation and analysis of disease spread dynamics and intervention strategies.

The results of simulations conducted under different conditions were presented, ranging from scenarios without intervention measures to those with lockdown measures and various approaches to vaccine and medicine distribution. These simulations showcased the framework’s versatility in exploring different intervention strategies and their potential impacts on disease control.

Overall, the framework provides a valuable tool for decision makers such as policy makers and health care professionals, enabling them to simulate and assess various intervention strategies based on social and economic factors. While operating under certain assumptions, the framework offers insights into disease control scenarios and aids in informed decision-making processes to develop optimal distribution plans for limited resources, such as vaccines and medications, and to evaluate the effectiveness of different intervention strategies.

The study presented some simulations but no implementation measures.

Among these simulations, there were the following:

- One that showed multiple waves of infection leading to a significant number of deaths and the immunization of certain individuals
- One that, through the application of confinement alongside the distribution of vaccines and medicines, shows the reduction in the spread of the disease
- One that revealed, that in the absence of intervention measures such as vaccines, medications, or lockdowns, the spread of infectious diseases can lead to significant morbidity and mortality, highlighting the importance of proactive intervention strategies
- One that indicated that targeted distribution strategies, such as prioritizing regions with higher infection rates, could potentially optimize vaccine effectiveness and minimize the overall disease burden

It would be interesting to compare the simulation results of lockdown, vaccine distribution, and medicine distribution. Such a comparison could provide valuable insights and potentially improve the overall outcome of this simulation.

This research is important because of the challenges epidemics and pandemics pose to public health systems and society as a whole. This model will help health agencies mitigate the spread in the event of an unexpected infection. The interdisciplinary approach and the breadth of scenarios explored make it an interesting contribution to the field of epidemic modeling and public health decision-making.

We would recommend that the study is tested in different geographic areas, with different distribution patterns. The distribution pattern may not be uniform from place to place. To ensure accurate results, it is also necessary to repeat the simulation using real-world data. The authors could consider collaborating with public health institutions to obtain data on the spread of disease. This could bring the simulations closer to reality and make their decision-making tool more effective and useful for users.

## List of Major Concerns and Feedback

- A significant issue with the study’s methodology is its dependence on simplifying assumptions. While simplifications are commonly used to make modeling more manageable, they can oversimplify intricate real-world dynamics, potentially causing inaccuracies in the simulation outcomes. For example, the assumption that everybody is the same is not feasible in a real-world setting; thus, this assumption may limit the applicability of the simulation in the real world. It is important to discuss the limitations of the simulations, such as simplifications and assumptions made in the modeling approach, as well as the potential implications of these limitations on the generalizability of the results.
- To ensure accurate results and to repeat the simulation using real-world data, consider collaborating with the Ministry of Health to obtain data on the spread of disease. Investigating practical implementation and real-time data integration could augment relevance and decision-making capacity in response to changing pandemic conditions.
- The central place theorem has a limited ability to evaluate the research question and handle dynamicity. It has unrealistic assumptions, shadow effects, and unexplained evolution. Please cite previous research on the usability of central place theory to model epidemics of infectious diseases. Also, consider exploring these two theories: central zones theory and urban resilience theory.
- What other techniques are available for distribution and how does the proposed model compare to them? How does this model fit into noninfectious diseases? Is it robust enough to be applied to other diseases?
- The study mentions adjusting parameters to match simulation outcomes with real-world data, but the process of validation and calibration needs to be more extensively discussed.
- Providing details on the criteria for initiating and lifting lockdown measures, as well as the impact of these measures on disease spread dynamics, would enhance the clarity of the discussion.
- The paper does not discuss conducting a sensitivity analysis. A sensitivity analysis aids in identifying which parameters have the greatest influence on the model outputs, guiding efforts to gather more precise data or refine model assumptions.
- Please clarify what the data sources were and whether ethical approvals were required to access the data and conduct the study. It would be important to understand how applicable the simulation is to a real-life pandemic situation, and therefore how usable it is. For example, what geographical areas were used in the simulation?
- Adding a justification for the selection of parameters in vaccine and medicine distribution modes would enrich the analysis. Clarifying how these choices correspond to real-world scenarios or theoretical frameworks would bolster the study’s strength.
- The authors could consider addressing whether the simulation is applicable for low-income countries where vaccine distribution may be challenging. It would be helpful to have more information on this matter.
- The authors should provide a detailed description of the disease’s clinical characteristics, highlighting any similarities it may have with human influenza or COVID-19. This is because simulations that work effectively for one disease may not necessarily work for another. Therefore, a thorough understanding of the disease’s unique clinical characteristics is crucial in developing accurate and effective simulation models.
- Analytically, the final meaning of the results is not reported. The Discussion section is not comprehensive enough.
- While the conclusions assert the framework’s capacity to simulate diverse scenarios and analyze various vaccine and medicine distribution strategies, a more comprehensive evaluation of the simulation results would enhance the discussion. This could entail examining the accuracy of the simulations in replicating real-world dynamics, juxtaposing the outcomes with historical data or alternative models, and pinpointing any constraints or avenues for enhancement. Such an approach would provide a deeper understanding of the framework’s effectiveness and offer valuable insights for future refinement.

## List of Minor Concerns and Feedback

- Please provide greater references to existing literature on how applicable the central place theory is in pandemic simulations and please include this in the Discussion.
- The study mentions adjusting parameters to match simulation outcomes with real-world data, but the process of validation and calibration needs to be more extensively discussed. Please include probability distribution definitions, specifications, and parameter details to ensure the reproducibility of the simulation. Statistics in supplementary material on how the transmission probability is calculated would also be useful.
- Furthermore, probability distribution definitions, specifications, and parameter details are missing, which are needed to be able to reproduce the simulation. The authors could consider providing more statistics in supplementary materials (eg, how is the probability of transmission calculated?).
- In the fourth paragraph of the Introduction, the authors should explicitly state that they are referring to a “new infection” because it might get confused with the previous discussion of COVID-19.
- No specific patient information is involved; the data is mostly imaginary. However, this study did not mention where they obtained the data from. So although ethical approval is not required, the authors should have mentioned this.
- Figures and tables:
  - The Results section seems bulky; the table could be avoided; instead of referring to the table, include the numbers in the text. Otherwise, if possible, relocate some of the figures to the appendix.
  - In the Features section, point 5, it would be helpful to use a diagram to explain the differences between the four vaccine/medicine distribution modes. The text is tricky to follow.
  - Provide thicker lines for the plots.
  - Indicate if the time steps are in actual time.
  - For simulation proportions, consider including the uninfected, sick, cured/immune, and dead population.
  - The graphs present numbers on the y-axis ranging between 800 and 1500, which can be considered a small population. In the Discussion section, indicate or comment on how the population size can be extrapolated to a city, town, or village.
  - Include lines for the total population on the graphs.
